# Correlation of the Rates of Solvolysis of *i*-Butyl Fluoroformate and a Consideration of Leaving-Group Effects

**DOI:** 10.3390/ijms12117806

**Published:** 2011-11-10

**Authors:** Yelin Lee, Kyoung-Ho Park, Mi Hye Seong, Jin Burm Kyong, Dennis N. Kevill

**Affiliations:** 1 Department of Chemistry and Applied Chemistry, Hanyang University, Ansan-si, Gyeonggi-do 426-791, Korea; E-Mails: redstar486@hanyang.ac.kr (Y.L.); piroho@hanyang.ac.kr (K.-H.P.); tpdlqk85@hanmail.net (M.H.S.); 2 Department of Chemistry and Biochemistry, Northern Illinois University, DeKalb, IL 60115-2862, USA; E-Mail: dkevill@niu.edu

**Keywords:** *i*-butyl fluoroformate, Grunwald-Winstein equation, leaving group effect, addition-elimination, solvolysis

## Abstract

The specific rates of solvolysis of isobutyl fluoroformate (**1**) have been measured at 40.0 °C in 22 pure and binary solvents. These results correlated well with the extended Grunwald-Winstein (G-W) equation, which incorporated the *N*_T_ solvent nucleophilicity scale and the *Y*_Cl_ solvent ionizing power scale. The sensitivities (*l* and *m*-values) to changes in solvent nucleophilicity and solvent ionizing power, and the *k_F_/k_Cl_* values are very similar to those observed previously for solvolyses of *n*-octyl fluoroformate, consistent with the additional step of an addition-elimination pathway being rate-determining. The solvent deuterium isotope effect value (*k_MeOH_/k_MeOD_*) for methanolysis of **1** was determined, and for solvolyses in ethanol, methanol, 80% ethanol, and 70% TFE, the values of the enthalpy and the entropy of activation for the solvolysis of **1** were also determined. The results are compared with those reported earlier for isobutyl chloroformate (**2**) and other alkyl haloformate esters and mechanistic conclusions are drawn.

## 1. Introduction

Linear free energy relationship (LFER) analysis with the extended Grunwald-Winstein (G-W) equation (Equation 1) has long been employed as a diagnostic tool for the study of solvent effects on solvolytic reactions [1,2].

(1) $$\text{log}\;(k/k_{\text{o}})={lN}_{\text{T}}+{mY}_{\text{Cl}}+c$$

In Equation 1,*k* and *k_o_* are the specific rates of solvolysis of a substrate in a given solvent and in 80% ethanol, respectively; *l* is the sensitivity towards changes in *N*_T_, a scale of solvent nucleophilicity based on the specific rates of solvolysis of *S*-methyl-dibenzothiophenium ion [3,4]; *m* is the sensitivity towards changes in *Y*_Cl_ [5–8], a scale of solvent ionizing power based on the specific rates of solvolysis of 1-adamantyl chloride, and *c* is the intercept.

(2) $$\begin{array}{c} \text{ROCOX}+2\text{SOH}\to \text{ROCOOS}+{{\text{SOH}}_{2}}^{+}+{\text{X}}^{-}\\ \text{ROCOOH}\to \text{ROH}+{\text{CO}}_{2}\;(\text{when S}=\text{H}) \end{array}$$

(3) (4)
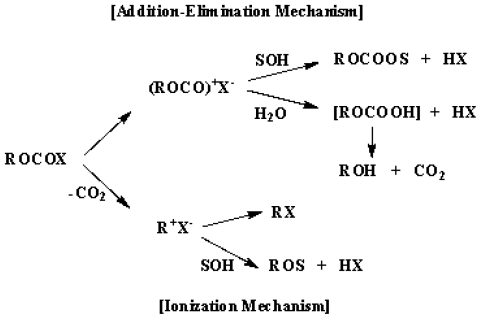


We have previously studied solvolysis reactions of alkyl and aryl haloformate esters concerning the application of the G-W equation [1,9]. In hydroxylic solvents, chloroformates with primary alkyl groups solvolyze with rate-determining attack at the carbonyl carbon (Equation 2). Only in solvents of very low nucleophilicity and very high ionizing power is an ionization mechanism observed (Equation 3) [10–13]. Secondary alkyl chloroformates follow the ionization pathway (Equation 4) in all the mixed solvents except for the more nucleophilic and less ionizing solvents (*i.e.*, in ethanol and methanol and their mixtures with 10% water) [14]. For tertiary 1-adamantyl chloroformate (1-AdOCOCl) [15], the ionization pathway (Equation 4) was dominant in all the mixed solvents and only in 100% ethanol was a trace of the mixed carbonate detected.

**Figure 1:**
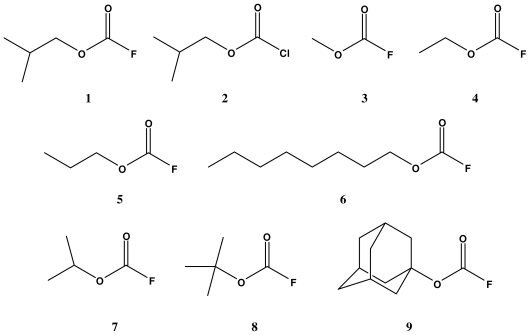
Molecular structures of isobutyl fluoroformate (**1**), isobutyl chloroformate (**2**), methyl fluoroformate (**3**), ethyl fluoroformate (**4**), *n*-propyl fluoroformate (**5**), *n*-octyl fluoroformate (**6**), isopropyl fluoroformate (**7**), tertiary butyl fluoroformate (**8**), and tertiary 1-adamantyl fluoroformate (**9**).

We have also reported that the solvolyses of primary and secondary alkyl fluoroformates (e.g., methyl (**3**) [16], ethyl (**4**) [17], *n*-propyl (**5**) [18], *n*-octyl (**6**) [19], and isopropyl (**7**) [20], Figure 1) in a wide range of hydroxylic solvents follow an addition-elimination pathway. Solvolyses of *t*-butyl fluoroformate (**8,** Figure 1) [21] were found to proceed entirely by an ionization pathway (Equation 4), which included the loss of carbon dioxide to give the relatively stable *t*-butyl cation. Solvolyses of tertiary 1-adamantyl fluoroformate (**9,** Figure 1) [22] led to two types of mechanisms, *i.e.*, a bimolecular pathway (Equation 2) and an ionization pathway (Equation 4). Accordingly, in order to extend the kinetic study on the solvolysis of alkyl haloformates to **1**, we have investigated the solvolysis reactions of **1** in a variety of pure and binary aqueous organic solvents using the extended G-W equation (Equation 1). We also report solvolysis studies at three different temperatures in four organic solvents to determine the values of enthalpy (*ΔH*^≠^) and entropy (*ΔS*^≠^) of activation and the solvent deuterium isotope effect for methanolysis (*k_MeOH_/k_MeOD_*). These analyses were combined with a consideration of leaving group effects, by comparison with the values for isobutyl chloroformate (**2**) solvolyses [13], to arrive at a reasonable mechanism.

## 2. Results and Discussion

The specific rates of solvolysis of **1** at 40.0 °C are reported in Table 1. The solvents consisted of ethanol, methanol, binary mixtures of water with ethanol, methanol, 2,2,2-trifluoroethanol (TFE), acetone (Me_2_CO), 1,1,1,3,3,3-hexafluoro-2-propanol (HFIP) and four binary mixtures of TFE and ethanol. The associated *N*_T_ and *Y*_Cl_ values are also reported in Table 1, together with the solvent deuterium isotope effect values for **1** and **2** in methanol-d (footnote to Table 1). The solvents studied include the important 2,2,2-trifluoroethanol (TFE)–water binary mixtures. These are important to minimize a multicollinearity which when powerful can invalidate the coefficients obtained by multiple regression analysis using extended forms of the Grunwald-Winstein equation. [23,24]. Specific rates of solvolysis of **1** were determined at four different temperatures in methanol, ethanol, 80% ethanol, and 70% TFE, and these values, together with calculated enthalpies and entropies of activation, are reported in Table 2. The obtained *l*, *m*, and *c* values, together with the corresponding literature values for solvolyses of other fluoroformate esters are summarized in Table 3.

As shown in Table 1, the specific rates for solvolysis of **1** increase with increasing water content in all the mixed solvents, indicating that the specific rate is accelerated by solvents with higher ionizing power (*Y*_Cl_). In contrast, solvolysis of **1** proceeds more rapidly with increasing ethanol content in the four TFE-ethanol solvents. These phenomena are similar to those previously reported for solvolysis of primary and secondary alkyl fluoroformates in various solvents [16–20], which have been suggested to proceed through an addition-elimination mechanism with the addition step being rate determining. As is the nature of the nucleophilic acyl substitution reactions, the rate of addition to the carbonyl carbon is slower as the steric demands of the alkyl group (*i.e.*, branching at the α-carbon) increase, and the rate of the reaction was enhanced as the electron withdrawing ability of the alkoxy group increases the positive charge on the carbonyl carbon (electronic effects) [25]. In Table 4, the order of the specific rate for solvolyses of **1** in relation to those previously studied for primary, secondary and tertiary alkyl fluoroformates is shown to be *k*_Me_ > *k*_Et_ ≈ *k*_n-Pr_ ≈ *k*_i-Bu_ ≈ *k*_n-Oct_ > *k*_i-Pr_ >*k*_t-Bu_ > *k*_1-Ad_ in 100% MeOH, 100% EtOH, and 80% EtOH but not in 70% TFE [16–22].

The difference in reactivity of **8** and **9** has previously been discussed by other workers [5,8]. The rate ratios for **1**, **4**, **5**, and **6** in 100% MeOH, 100% EtOH, and 80% EtOH (Table 4) being close to unity (*k*_ROCOF_/*k*_EtOCOF_ = 0.91~1.15) suggests that electronic and/or steric influences due to the presence of a branching β-alkyl group in the alkyl fluoroformates can be neglected. On the other hand, the specific rate of solvolysis of **8** in 70% TFE was somewhat faster than the rates of **1**, **4**, **5**, **6** and **7**. The higher rate ratio (*k*_t-BuOCOF_/*k*_EtOCOF_ = 5.6) in 70% TFE relative to 100% MeOH, 100% EtOH, and 80% EtOH probably results from solvolysis of **8** (via the relatively stable t-butyl cation) being more favored by the electrophilic influence of the relatively acidic TFE than the other alkyl fluoroformates, which are believed to proceed by a bimolecular pathway, *i.e.*, for tertiary alkyl haloformates that undergo solvolysis by an ionization process, an increase in the polarity of the solvent and/or its ion-solvation ability resulted in a significant increase in reaction rate. The specific rate of solvolysis of **8**, which has been reported to proceed through an ionization pathway, was found to be 4.0 × 10^2^ times faster in 70% TFE (*Y*_Cl_ = 2.96) than in 100% EtOH (*Y*_Cl_ = −2.52). This phenomenon occurs because, in the ionization pathway, charge is developed and concentrated in the transition state compared with the starting material (Equation 4).

Consideration of leaving group effects in nucleophilic substitution reactions has long been recognized as a useful tool in studying reaction mechanisms [26]. For the ionization pathway, a value of *k*_F_/*k*_Cl_ = 1.3 × 10^−4^ was observed for acetyl halide solvolyses [27], and a low value of *k*_F_/*k*_Cl_ = 1.20 × 10^−5^ − 3.17 × 10^−5^ was observed for 1-adamantyl haloformate solvolyses [15,22]. Since the carbon-fluoride bond (C-F) is much stronger than the carbon-chloride bond (C-Cl), if the carbonhalogen bond is broken in the rate-determining step (ionization pathway), the *k*_F_/*k*_Cl_ ratio would be expected to exhibit a significant leaving group effect, *k*_F_ ≪ *k*_Cl_. In contrast, if the addition step is ratedetermining (*i.e.*, bimolecular pathway), values of close to unity, reflecting a large electron deficiency at the carbonyl carbon of the fluoroformate, would be frequently observed, and the bimolecular pathway through a tetrahedral intermediate formed by the rate-determining addition of solvent to the carbonyl carbon would be characterized by *k*_F_ ≥ *k*_Cl_. A previous report concerning the solvolyses of **4**, **5**, **6** and **7** [17–20], which were believed to solvolyze by an addition-elimination mechanism, found a *k*_F_/*k*_Cl_ ratio to be somewhat below unity in ethanol and methanol and slightly greater than unity for solvolyses in mixtures of water with ethanol, methanol, acetone, or TFE (Table 5). For other haloformate esters, *k*_F_/*k*_Cl_ ratios of 1.09 to 7.16 for solvolyses in 70% aqueous acetone at 30.1 °C have been reported [28]. As mentioned above, for binary solvents, the specific rates for solvolysis of the fluoroformate are somewhat faster, despite the stronger carbon-fluorine bond. As shown in Table 5, the *k*_F_/*k*_Cl_ ratios were similar for all the substrates, which have been reported to proceed through a bimolecular pathway. They are smaller for the 1-adamantyl substrates, which presumably proceed through a solvolysis-decomposition reaction (ionization pathway) in ethanol, methanol, and 80% ethanol.

The solvent deuterium isotope effect value for methanolysis of **1** is of a magnitude usually taken to indicate that nucleophilic attack by a methanol molecule is assisted by general-base catalysis by a second methanol molecule (Table 4) [29–31]. The value (*k*_MeOH_/*k*_MeOD_ = 3.40 at 40.0 °C) for **1** is slightly larger than for methanolysis of **2** (*k*_MeOH_/*k*_MeOD_ = 2.00 at 40.0 °C), which further supports the proposal that bond formation is more advanced in the transition state for addition to fluoroformate than for chloroformate. The solvent deuterium isotope effect has previously been studied for several solvolyses of haloformate esters (Table 4). In methanol, the *k*_MeOH_/*k*_MeOD_ ratio was in the range of 2.00 to 3.98 for solvolyses of alkyl and aryl haloformates which have been reported to proceed through a bimolecular mechanism [10–13,16–20,32,33]. The *k*_ROH_/*k*_ROD_ values for *i*-propyl chloroformate and **8** in the range of an ionization mechanism, were somewhat smaller at 1.25 in pure water [34] and 1.26 [21] in methanol, respectively.

For solvolyses in ethanol, methanol, 80% ethanol, and 70% TFE, the values of the enthalpy and the entropy of activation for the solvolysis of **1** (Table 2) are 9.5~11.1 kcal·mol^−1^ and −45.3~−39.9 cal·mol^−1^·K^−1^, respectively. The large negative entropies of activation observed for **1** in the four solvents are consistent with the bimolecular nature of the rate-determining step. The mechanism for the solvolysis of **1** is similar to that reported for the solvolyses of **3**, **4**, **5**, **6** and **7** in several solvents, which have been suggested to proceed through a bimolecular pathway [16–20].

**Figure 2:**
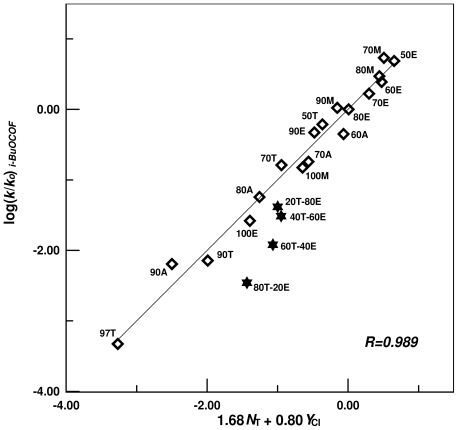
Plot of log (*k/k_o_*) for solvolyses of isobutyl fluoroformate (**1**) at 40.0 °C against (1.68*N*_T_ + 0.80*Y*_Cl_). The log (*k/k_o_*) values for the four TFE-EtOH mixtures are not included in the correlation; they are added to show their moderate deviation from the correlation line.

The extended G-W Equation 1 gives information which is very helpful in assessing the mechanism of solvolysis reactions. Therefore a correlation analysis of the specific rates for the solvolysis of **1** was carried out using the extended G-W equation 1 and the *l* and *m* values were compared with those previously obtained from the solvolyses of other alkyl haloformates (Table 3) [13,16–22]. As shown in Figure 2, inspection of the plot corresponding to this correlation showed that the four data points for solvolyses in TFE-ethanol mixtures show moderate to appreciable deviation from the linear plot. This was previously discussed in detail using the extended G-W Equation 1 to the specific rates of solvolysis of alkyl and acyl haloformate esters in binary TFE-ethanol solvents [14,22,35,36] and will not be considered again in this report. Correlations were carried out both with and without the TFE-ethanol data, and analysis of the data obtained applying the extended G-W equation 1 to the specific rates of solvolysis of **1** in all the solvents led to an acceptable linear correlation with values of 1.78 ± 0.13 for *l,* 0.85 ± 0.10 for *m*, −0.07 ± 0.10 for *c*, and 0.956 for the correlation coefficient. Recalculation with omission of these TFE-ethanol mixture data led to a very good linear correlation with values of 1.68 ± 0.07 for *l*, 0.80 ± 0.04 for *m*, 0.01 ± 0.05 for *c*, and 0.989 for the correlation coefficient.

The relative magnitudes of *l* and *m* (*l*/*m* ratio) have often been suggested to be useful mechanistic criteria. As shown in Table 3, the *l*/*m* ratio clearly is divided into two classes with values of 1.8~2.8 for those entries postulated to be associated with the addition-elimination (A-E, Equation 2) mechanism and values of 0.54~0.84 for those believed to be associated with the ionization (I, Equations 3 and 4) mechanism. Table 3 shows that the *l* and *m* values of **1** are similar to those previously reported for other primary and secondary alkyl fluoroformates, e.g., a plot of log (*k*/*k*_o_) for **1** against log (*k*/*k*_o_) for **6** shows a good linear correlation [*i.e.*, log (*k*/*k*_o_)_i-butyl_ = 0.98 log (*k*/*k*_o_)_n-octyl_ + 0.09, R = 0.990] in pure and mixed solvents. A good linear relationship for the solvolyses of **1** and **6** provides strong evidence for a similarity model. The higher *m*-value for the solvolyses of **1**, relative to **2**, may reflect the kinetically favorable influence of increased solvation of the developing negative charge on the carbonyl oxygen in the presence of the more electronegative fluorine attached at the carbonyl carbon [16–20,37,38]. As shown in Table 3, the solvolyses of primary and secondary alkyl fluoroformates [methyl (**3**), ethyl (**4**), *n*-propyl (**5**), *n*-octyl (**6**), *i*-butyl (**1**) and *i*-propyl (**7**)] in all the solvents studied were found to proceed through only an addition-elimination mechanism with the addition step being rate-determining (Equation 2), despite the increasing chain length in primary alkyl fluoroformates (**1**, **3**, **4**, **5** and **6**) and the influence of a branched-chain alkyl group as in **7**.

## 3. Experimental Section

Isobutyl chloroformate (**2**, Aldrich, 21.06 g, 0.154 mol) was syringed into a three-neck flask containing dried KF (11.5 g, 0.198 mol) and 18-crown-6 (1.44 g, 0.0054 mol) and fitted with a teflon stirring bar, a condenser topped by an N_2_ gas inlet, a septum cap, and a ground glass stopper, as described previously [20,39]. The mixture was stirred efficiently at room temperature until IR analysis of an aliquot indicated that no chloroformate remained (C=O stretch at 1779 cm^−1^; fluoroformate C=O stretch at 1830 cm^−1^). After a few more hours (total reaction time 45 h), isobutyl fluoroformate (**1**) was isolated directly from the reaction apparatus by simple distillation: yield 17 g (92%); b.p. 90–92 °C at atmospheric pressure (b.p. 92–93 °C at atmospheric pressure [40]).

Solvents used in solvolysis experiments were purified as previously described [13]. All runs were performed using a substrate concentration of 5.81 × 10^−3^ mol dm^−3^, and 5 mL portions were removed for titration, except for runs in TFE-H2O and TFE-EtOH mixtures in which 2 mL portions were used for titrations. Due to the faster rates for the solvolysis of 1 in 50% EtOH and 70% MeOH than in other solvents, these kinetic measurements were made conductometrically using a Metrohm 712 (Swiss), with an immersion measuring cell (Pt 100). Runs were carried out in duplicate with for each run at least 95 readings for conductivity measurements and eight to ten readings for titration measurements, with infinity readings after ten half-lives. All the integrated specific rate values obtained from each run for a given solvolysis were used to calculate an overall average value and the associated standard deviation. The l and m values were calculated using multiple regression analysis.

## 4. Conclusions

The solvolyses of **1** give a satisfactory extended Grunwald-Winstein correlation over a wide range of *N*_T_ and *Y*_Cl_ values. The sensitivities to changes in *N*_T_ and *Y*_Cl_ (*l* = 1.68 and *m* = 0.80) are similar to those for several alkyl fluoroformates, which are shown to solvolyze by an addition-elimination pathway with the addition step being rate determining. The *k*_F_/*k*_Cl_ values obtained in a comparison with the corresponding solvolysis of **2** are similar to those for the solvolyses of **3**, **4**, **5**, **6**, and **7**, consistent with a bimolecular addition-elimination mechanism, proceeding through a tetrahedral intermediate. The solvent deuterium isotope effect value (*k*_MeOH_/*k*_MeOD_ = 3.40) for methanolysis of **1** is of a magnitude usually taken to indicate that nucleophilic attack by a methanol molecule is assisted by general-base catalysis by a second methanol molecule. The large negative entropies of activation (−45.3~−39.9 cal·mol^−1^·K^−1^) observed for the solvolyses of **1** in four solvents are consistent with the bimolecular nature of the ratedetermining step. In the present study, unlike the solvolyses of **2**, where two reaction channels were observed (*i.e.*, the addition-elimination pathway, Equation 2 and the ionization pathway, Equation 4), the solvolyses of **1** proceed through only an addition-elimination pathway (Equation 2) with the addition step being rate-determining.

## Figures and Tables

**Table 1:** Specific rates of solvolysis of isobutyl fluoroformate (**1**) *_a_* in pure and binary solvents at 40.0 °C and the *N*_T_ and *Y*_Cl_ values for the solvents.

Solvent b	10^4^*k* (s^−1^) c	*N*_T_^d^	*Y*_Cl_^d^
100% MeOH e	2.15 ± 0.02 g	0.17	−1.17
90% MeOH	15.1 ± 0.2	−0.01	−0.18
80% MeOH	42.5 ± 0.2	−0.06	0.67
70% MeOH	77.6 ± 0.2	−0.40	1.46
100% EtOH	0.378 ± 0.002	0.37	−2.52
90% EtOH	6.77 ± 0.04	0.16	−0.94
80% EtOH	14.4 ± 0.1	0.00	0.00
70% EtOH	24.1 ± 0.1	−0.20	0.78
60% EtOH	35.3 ± 0.2	−0.38	1.38
50% EtOH	70.0 ± 0.2	−0.58	2.02
90% Acetone	0.0923 ± 0.0002	−0.35	−2.39
80% Acetone	0.823 ± 0.001	−0.37	−0.80
70% Acetone	2.60 ± 0.01	−0.42	0.17
60% Acetone	6.44 ± 0.03	−0.52	1.00
97% TFE	0.00678 ± 0.00005	−3.30	2.83
90% TFE	0.103 ± 0.003	−2.55	2.85
70% TFE	2.33 ± 0.04	−1.98	2.96
50% TFE	8.81 ± 0.04	−1.73	3.16
80T-20E f	0.0500 ± 0.0005	−1.76	1.89
60T-40E f	0.173 ± 0.002	−0.94	0.63
40T-60E f	0.440 ± 0.002	−0.34	−0.48
20T-80E f	0.598 ± 0.004	0.08	−1.42

a Substrate concentration is 5.81 × 10^−3^ mol dm^−3^;

b Volume/volume basis at 25.0 °C, except for TFE–H_2_O mixtures, which are on a weight/weight basis;

c The average of all integrated specific rates from duplicate runs, with associated standard deviation;

d From references [4] and [6];

e Specific rates of solvolysis of isobutyl chloroformate (**2**) in 100% MeOH and 100% MeOD at 40.0 °C are (3.27 ± 0.05)_MeOH_ × 10^−4^ s^−1^ and (1.64 ± 0.02)_MeOD_ × 10^−4^ s^−1^, respectively, and the *k*_MeOH_/*k*_MeOD_ value of solvolysis of isobutyl chloroformate is 2.00 ± 0.02;

f T-E are 2,2,2-trifluoroethanol-ethanol mixtures;

g Value in MeOD of 0.632 ± 0.003, and a solvent deuterium isotope effect (*k*_MeOH_/*k*_MeOD_) of 3.40 ± 0.02.

**Table 2:** Specific rates for solvolysis of isobutyl fluoroformate (**1)** at various temperatures and enthalpies (*ΔH*^‡^, kcal·mol^−1^) and entropies (*ΔS*^‡^, cal·mol^−1^·K^−1^**)** of activation.

Solvent a	T, °C	10^4^*k* (s^−1^) b	*ΔH*^‡^*_313_*^c^	*ΔS*^‡^*_313_*^c^
100% MeOH	40.0	2.15 ± 0.02 d	9.5 ± 0.2	−45.2 ± 0.7
	45.0	2.80 ± 0.04		
	50.0	3.51 ± 0.02		
	55.0	4.55 ± 0.02		
100% EtOH	40.0	0.378 ± 0.002 d	10.5 ± 0.2	−45.3 ± 0.8
	45.0	0.508 ± 0.002		
	50.0	0.671 ± 0.002		
	55.0	0.854 ± 0.003		
80% EtOH	40.0	14.4 ± 0.1 d	9.8 ± 0.5	−40.3 ± 1.5
	45.0	19.5 ± 0.2		
	50.0	25.0 ± 0.2		
	55.0	31.0 ± 0.3		
70% TFE	40.0	2.33 ± 0.04 d	11.1 ± 0.4	−39.9 ± 1.4
	45.0	3.08 ± 0.01		
	50.0	4.03 ± 0.04		
	55.0	5.54 ± 0.02		

a,b See footnotes in Table 1;

c With associated standard error;

d From Table 1.

**Table 3:** Correlations of the specific rates of solvolysis of isobutyl fluoroformate (**1**) and a comparison with the corresponding values for the solvolyses of isobutyl chloroformate and other fluoroformates using the Grunwald-Winstein equation.

Substrate	Mech.^a^	n b	*l*^c^	*m*^c^	*c*^c^	*R*^d^	*l/m*
**1**	A-E	22 e	1.78 ± 0.13	0.85 ± 0.10	−0.07 ± 0.10	0.956	2.09
	A-E	18 e,f	1.68 ± 0.07	0.80 ± 0.04	0.01 ± 0.05	0.989	2.10
**2**	A-E	18 g	1.82 ± 0.15	0.53 ± 0.05	0.18 ± 0.07	0.957	3.43
**3**	A-E	14 h	1.33 ± 0.09	0.73 ± 0.06	−0.08 ± 0.08	0.972	1.82
**4**	A-E	17 i	1.34 ± 0.14	0.77 ± 0.07	−0.06 ± 0.10	0.942	1.74
**5**	A-E	16 j	1.72 ± 0.12	0.91 ± 0.08	0.05 ± 0.08	0.970	1.89
**6**	A-E	19 k	1.67 ± 0.07	0.76 ± 0.03	−0.08 ± 0.18	0.988	2.20
**7**	A-E	20 l	1.59 ± 0.16	0.80 ± 0.06	0.06 ± 0.08	0.957	1.99
8	I	17 m	0.41 ± 0.05	0.65 ± 0.03	0.02 ± 0.04	0.989	0.63
**9**	A-E	10 n	2.78 ± 0.21	1.01 ± 0.06	0.09 ± 0.16	0.987	2.78
	I	16 n	~0	0.70 ± 0.01	−0.02 ± 0.05	0.999	~0

a Addition-elimination (A-E) and ionization (I);

b Number of solvent systems included in the correlation;

c Using equation 1, with standard errors for *l* and *m* values and with standard errors of the estimate accompanying the *c* values;

d Correlation coefficient;

e This study;

f Omitting the TFE-ethanol solvents;

g The solvent systems with omission of the four TFE-H_2_O solvents, reference [13];

h Reference [16];

i Reference [17];

j Reference [18];

k Reference [19];

l Reference [20];

m Reference [21];

n The 26 solvent systems divided into 16 aqueous fluoroalcohol solvents and the remainder (reference [22]).

**Table 4:** A comparison of the specific rates (10^4^ *k* (s^−1^)) ^a,b^ of solvolysis of several alkyl fluoroformates (ROCOF) in pure and binary solvents at 25.0 °C, and the solvent isotope effect values (*k*_MeOH_/*k*_MeOD_).

Solvent c	methyl e	ethyl f	*n*-propyl g	*i*-butyl h	*n*-octyl i	*i*-propyl j	*t*-butyl k	1-adamantyl l
100% MeOH	2.47 (5.81)	0.836 (2.32)	- (2.19)	0.952 (2.15)	0.853	0.217	7.43 × 10^−2^	2.51 × 10^−2^
100% EtOH	0.424 (1.09)	0.135 (0.394)	- (0.437)	0.155 (0.378)	0.153	3.93 × 10^−2^	1.31 × 10^−2^	2.29 × 10^−3^
80% EtOH	19.1 (43.6)	6.52 (14.3)	- (14.0)	6.31 (14.4)	5.96 m	1.71	0.616	0.150
70% TFE d	4.75 (10.8)	1.23 (3.61)	- (2.20)	0.895 (2.33)	0.430 m	0.240	6.84	-
*k_MeOH_/k_MeOD_*	3.98 e	3.10 f	3.32 g	3.40 h	-	2.53 j	1.26 k	-

a Values obtained using Arrhenius plots with the specific rates reported at different temperatures;

b Values in parentheses represent the specific rates obtained at 40.0 °C;

c Unless otherwise indicated, on a volume/volume basis, at 25.0 °C, with the other component water;

d Solvent prepared on weight/weight basis;

e From reference [16];

f From reference [17];

g From reference [18];

h This study;

i From reference [19];

j From reference [20];

k From reference [21];

l 1-adamantyl fluoroformate, from reference [22];

m Values at 24.2 °C.

**Table 5:** The specific rate ratios (*k_F_/k_Cl_*) of solvolyses of alkyl haloformates in pure and binary solvents at various temperatures.

Solvent a	methyl c	ethyl d	*n*-propyl e	*i*-butyl f	*n*-octyl g	*i*-propyl h	1-adamantyl i
100% EtOH	0.83	0.57	0.57	0.45	0.62	0.18	1.20 × 10^−5^
80% EtOH	8.28	8.74	5.62	5.43	8.09	2.11	3.17 × 10^−5^
60% EtOH	-	14.0	-	8.43	15.1	1.79	-
100% MeOH	1.12	0.93	0.75	0.66	0.95	0.39	1.56 × 10^−5^
90% MeOH	5.11	4.82	-	2.42	-	1.76	-
80% Me_2_CO	3.71	3.90	4.24 j	2.60	2.86	0.53	-
70% TFE b	27.2	19.3	7.72	8.86	10.2 k	0.067	-

a Unless otherwise indicated, on a volume/volume basis, at 25.0 °C, with the other component water;

b Solvents prepared on weight/weight basis;

c At 40.0 °C in reference [16];

d At 24.2 °C in reference [17];

e At 40.0 °C in reference [18];

f At 40.0 °C in this study;

g At 24.2 °C in reference [19];

h At 40.0 °C in reference [20];

i At 50.0 °C in reference [22];

j For 70% Me_2_CO;

k For 80% TFE.
